# Real-Time Locating Systems and the Effects on Efficiency of Anesthesiologists

**Published:** 2018-03-29

**Authors:** Cindy Yeoh, Jennifer Mascarenhas, Kay See Tan, Luis Tollinche

**Affiliations:** Department of Anesthesiology and Critical Care Medicine, Memorial Sloan Kettering Cancer Center, New York, USA

## Abstract

**Objective:**

To investigate the impact of Real Time Locating System (RTLS) technology on the perioperative efficiency of anesthesiologists.

**Methods:**

A retrospective chart review was performed for all outpatient and short-stay patients who received General Anesthesia care at our institution between January 2016 and October 2017. Patients included were over 18 years and had ASA classification scores of 1, 2, and 3. Only first cases of the day for individual anesthesiologists were included. Duration between two perioperative time points was collected and used as a measure of efficiency. Two groups of anesthesiologists were compared

**Group 1:** Anesthesiologists at Main Campus who do not use RTLS
**Group 2:** Anesthesiologists at Josie Robertson Surgery Center who use RTLS

The outcome measure collected from patient electronic medical records was defined as

**DUR:** Duration from when patient is admitted to the operating room and initiation of induction only for first case of the day by attending anesthesiologist. The outcome was compared between the two groups using Wilcoxon rank sum test.

**Results:**

The duration between admission to the OR and initiation of induction was significantly shorter in JRSC (with RTLS) than main campus (without RTLS); specifically, median (25^th^, 75^th^ percentile) of the duration was 7.0 (5.0, 10.0) at JRSC vs. 8.0 (6.0, 11.0) at main campus (p < 0.0001, Table 1).

**Conclusion:**

In our initial study, we found that anesthesiologists who had access to RTLS at JRSC performed more efficiently in their preoperative evaluation of patients as well as time to induction for general anesthesia cases. Because of various confounding factors that potentially influenced the increase in efficiency of anesthesiologists with access to RTLS, this follow-up study aims to eliminate several confounding factors by assessing only time to induction of general anesthesia for all first cases of the day by anesthesiologists. We continue to find a small yet statistically significant difference in time to induction of anesthesiologists with access to RTLS. This translates directly into increased efficiency in perioperative workflow. Additional investigation and application can help elucidate the true value of RTLS on workflow efficiency in the healthcare setting.

## Introduction

Operating room staff aim to improve their efficiency and streamline workflow to meet the demands of rising patient census levels and increased healthcare costs [1]. Many healthcare facilities in the United States are in active pursuit of technologies and systems that can aid in improving workflow and efficiency. Real-time locating system (RTLS) is a technology that is able to provide immediate or real-time tracking and management of medical equipment, staff and patients within all types of healthcare settings [2]. Originally used to track and locate medical equipment, RTLS technology provides and stores location and time-specific data, e.g. when a patient enters the pre-surgical area, when a physician interacts with the patient, and when a patient enters the operating room [3–5]. While positive applications have been widely supported with data on the tracking of hospital assets, few studies have addressed the effects of RTLS on the efficiency and productivity of healthcare providers [6]. Because the original intent of RTLS was for tracking medical equipment and not professional healthcare providers, there are privacy and autonomy concerns regarding its use in this context that will be discussed.

Increased operating room (OR) efficiency ideally equates to shorter OR times and decreased OR turnover time which leads to less patient wait times, improved patient experiences, and a decrease in valuable OR time and resources. Given the concentration of specialized physicians and the state of the art equipment and technology in current ORs, each minute of OR time is valued between $30–80/min, rendering the OR as the most expensive unit in a hospital [7–9]. In addition to delivering safe patient care, the anesthesia team is a critical component of ensuring efficiency during the perioperative period [10,11].

In January of 2016, our institution opened a free standing ambulatory surgical center (Josie Robertson Surgery Center, JRSC) with cutting edge technology, including RTLS. RTLS was implemented for the purpose of facilitating perioperative efficiency and workflow. Because our Main Campus does not have RTLS technology, we investigated if there was a difference in efficiency of anesthesiologists who have access to RTLS at JRSC versus anesthesiologists who do not have access to RTLS at Main Campus.

In our initial study, we investigated two time points during the anesthesiologist’s workflow to determine if any difference exists in efficiency between those who have access to RTLS (JRSC) and those who do not (Main Campus). We found that anesthesiologists who utilize RTLS were consistently faster, and hence more efficient in their perioperative workflow [12]. While our results were statistically significant, there were confounding factors which could have accounted for the difference in perioperative efficiency. In this follow-up study, we attempt to address and eliminate confounding factors by evaluating one primary outcome measure of efficiency: Duration (in minutes) between when a patient is admitted to the OR and initiation of induction of first general anesthesia (GA) cases of the day by anesthesiologists.

## Methods

We performed a retrospective chart review for all outpatient and short-stay patients who received GA at both JRSC and Main Campus at our institution between January 2016 and October 2017. Only first cases of the day for all anesthesiologists were included in this study. The rationale behind this was to eliminate the confounding factor of an anesthesiologist being busy with a patient in a preoperative evaluation, or starting another case in a different OR that would result in a delay and increase in duration arriving for induction. To ensure comparable patients between the two groups, other inclusion criteria were patients over 18 years, GA cases for all surgical sub-specialties, outpatient and short stay cases, and ASA classification of 1, 2, and 3. ASA 4 and 5 patients were excluded to eliminate the possibility that higher acuity patients may have a longer pre-induction time (“OR arrival to induction time”) due to reasons such as placement of pre-induction arterial line etc. OR staffing was comparable at both locations. Like our previous study, time was used as a measure of efficiency between the two groups of anesthesiologists.

The two comparison groups are as follows:

Group 1: Anesthesiologists at our institution’s Main Campus who do not have access to RTLS technology
Group 2: Anesthesiologists at JRSC where RTLS is available and its use is mandatory.

In this follow-up study, only one primary outcome measure was collected from patient electronic medical records:

DUR: Duration (in minutes) between when a patient is admitted to the operating room and initiation of induction by attending anesthesiologist. The primary outcome was compared between the two groups using Wilcoxon rank sum test. Analyses were conducted with Stata 13.1 (StataCorp, College Station, TX). All statistical tests were two-sided and p < 0.05 is considered significant.

## Results

Through retrospective chart review of electronic patient records, 5395 records matched inclusion criteria of GA cases for patients over 18 years that were first case of the day (all case types for all surgical sub-specialties) at Main Campus (N = 2890) and JRSC (N = 2505). Of these, 3158 were outpatient versus 2237 short stay cases. ASA classifications for all included cases were: ASA 1 (N = 117), ASA 2 (N = 2301), and ASA 3 (N = 2977).

For our primary outcome measure, the duration between admission to the OR and initiation of induction was significantly shorter in JRSC (with RTLS) than main campus (without RTLS); specifically, median (25^th^, 75^th^ percentile) of the duration was 7.0 (5.0, 10.0) at JRSC vs. 8.0 (6.0, 11.0) at Main Campus (p < 0.0001, Table 1).

## Discussion

There are several time points in the perioperative period that involve anesthesiologists, where improved efficiency may translate to a decrease in operating room time and reduced healthcare costs. Our previous study concluded that anesthesiologists at our institution with access to RTLS were significantly more efficient in completing their preoperative anesthesia evaluation (p < 0.0001) than those without access to RTLS [12]. This follow- up study attempts to eliminate variability and focuses on the time difference between when a patient enters the OR to the time of induction for only the first cases of the day between anesthesiologists without (Group 1) and with RTLS (Group 2). We demonstrated that Group 2 was one minute more efficient overall with time to induction versus Group 1 (p < 0.0001). These results are consistent with findings from our initial study. Anesthesiologists with access to RTLS were found to be more efficient in their preoperative patient evaluation and time to anesthesia induction [12].

Access to RTLS may decrease time to induction and improve efficiency in the following ways:

- It gives all OR staff the ability to track the location of the entire OR team, including the patient and anesthesiologist, which may allow for a more efficient process in deeming an OR “ready”. Perhaps members of the OR support staff, such as OR nurses and certified registered nurse anesthetists (CRNAs), view the location of the Anesthesiologist on the RTLS computer application and notify them that the patient is on the way to the OR.
- Hawthorne effect: Healthcare providers may display behavior modifications in response to their awareness of being observed and monitored. Some changes in behavior may improve perioperative workflow and efficiency. The awareness that one is being tracked and monitored may motivate all OR staff, including anesthesiologists, to be readily available once the OR is deemed “ready” [13].

Despite these findings, the benefits of RTLS such as positive behavior modification resulting in improved workflow must be balanced with ethical and legal concerns of continuous location tracking. Originally designed to track and monitor medical equipment and supplies, continuous location monitoring of healthcare workers is complicated, and few studies have addressed the effects of RTLS on the efficiency and productivity of healthcare providers. RTLS systems allow for live, minute- to-minute tracking; however, many systems also record and store information indefinitely. This constant “eye” can be unsettling to healthcare providers as their time spent with every patient is recorded. It is unknown if stored RTLS data can be requested in a medical-legal case; the legal implications are vast [14–16].

Another issue with RTLS is the lack of consent for continuous location tracking. Patients, family members and healthcare providers who are given an RTLS badge in a healthcare setting are automatically opted in for location tracking; however, none are asked for their consent. Currently, the right of an employee to location privacy has not been clearly established in the United States. At the time of this writing, there are no laws which directly address employee location monitoring in the US which leaves employers with considerable latitude to monitor their workers as an extension of the right to control workflow [14–16].

RTLS technology allows hospital administration to monitor many aspects of their healthcare providers’ workplace activities and can potentially provide insight into their staff’s behavior based on the digital footprint trail created by RTLS. This digital information may be used to evaluate staff performance and conduct. Concerns regarding employee, family, and patient privacy need to be considered and addressed with use of RTLS in the healthcare setting.

## Conclusion

This follow-up RTLS study assessed the duration between when a patient entered an OR and time of induction for the first case of the day between JRSC where all staff is required to wear RTLS versus Main Campus where RTLS is not implemented. This study found a significant decrease in time to induction by the anesthesiologists who wear RTLS. This decrease in time to induction decreases OR time and cost, and increases OR workflow efficiency. Additional cost-effective analyses need to be undertaken to assess the value of this increase in perioperative efficiency, especially considering ethical and legal concerns surrounding electronic tracking of employees in the workplace.

## Figures and Tables

**Table 1:** Duration (in minutes) between admission to the OR and initiation of induction.

	Main Campus (N = 2890; 54%)	JRSC (N = 2505; 46%)	p
DUR	8.0 (6.0, 11.0)	7.0 (5.0, 10.0)	< 0.0001

DUR: Duration (in minutes) between when patient is admitted to the operating room and initiation of induction by the attending anesthesiologist. Values are presented as median (25^th^, 75^th^ percentile).
